# Supplementary material for: Are total omega-3 and omega-6 polyunsaturated fatty acids predictors of fatal stroke in the Adventist Health Study 2 prospective cohort?
Source: PLoS One. 2022 Sep 9;17(9):e0274109. doi: 10.1371/journal.pone.0274109 (PMC9462555; doi:10.1371/journal.pone.0274109)
Supplement: Attachment — Submitted filename: ResponseToReviewers.docx [file pone.0274109.s001.docx]

**REVIEWER 1:**

Abstract: Please include the main objective of the study

Thank you for your feedback. To highlight the objective of the study, we added the following new line to the abstract introduction: “This study therefore intended to investigate these effects in a cohort for which the vegetarian diet was more prevalent than the general public.”

Methods: Please provide detailed information about supplements intake.

To clarify the nature of the supplements, we added two new sentences to the Methods section under the Study Variables header: “Approximately 12% of participants supplemented n-3 PUFA, and approximately 15% supplemented n-6 PUFA. However, overall rates of supplementation were low: the 90th percentile of n-3 PUFA supplement contribution was 8.5% of total n-3 PUFA intake, and the corresponding 90th percentile of total n-6 PUFA intake due to n-6 PUFA supplements was 0.1%.”

Discussion: For discussion the 10th percentile (6.1:1) found in this study (percentile with lower risk of fatal stroke), suggest to the authors add a comment about the n-6/n-3 PUFA ratio found in studies with the Mediterranean Diet (diet known for the protection in cardiovascular and cerebrovascular events) or with the n-6/n-3 PUFA ratio found in longevity studies.

To improve our discussion about the n-6/n-3 PUFA ratio, we added a new sentence in the Discussion section to describe other populations’ n-6/n-3 PUFA ratios: “A previous review has investigated the n-6 / n-3 PUFA ratio of various diets and noted that while the present Western diet has an n-6 / n-3 PUFA ratio of 15-20:1, other populations have much lower ratios. Examples include Japan at 4:1 and rural India at 5-6:1, and specific diets such as Swedish at 4.7:1 or Mediterranean at 2.6:1 (52).”

**REVIEWER 2:**

Methods - What do the authors mean by this sentence, "The AHS-2 has been described in detail elsewhere..."? I suggest indicating proper where all the information has been described.

Thank you for this suggestion. We used this formal shorthand to denote that a separate journal article exists wherein the study is described in extensive detail. Recent PLOS One articles which have utilized similar statements include DOI=10.1371/journal.pone.0270033 (“Additional details are described elsewhere [18].”), DOI=10.1371/journal.pone.0269042 (“The detailed study design has been previously described [28].”), and DOI=10.1371/journal.pone.0270893 (“The study utilized MHS pregnancy registry, previously described [13].”). For improved clarity, we have updated the text to now read: “The AHS-2 has been described in detail previously by Butler et. al.”

Methods- It is unclear how the authors investigated in the same category participants that had limited or no current consumption of tobacco and alcohol. It seems to me that they should be split into different groups.

Historic tobacco and alcohol use were low in this cohort, so categories of use were collapsed. For clarity, we have added the following to a sentence in the Methods section describing tobacco and alcohol rates: “so variables controlling for those factors were collapsed”

Methods- I could not find the description appropriate to the acronym BMI. Even if it's a common acronym, it must be defined the first time it is included in the text.

The definition of BMI was added for the first use of the term, in the Methods section: “or body mass index (BMI).”

Table 1 - What do the authors mean by the smoking ever used? Participants smoked in the past? For how long? How many cigarettes a day? For how long did they stop smoking? Please clarify.

As previously mentioned, historic tobacco use was very low in this cohort so categories were collapsed. For clarification, the Methods section has now been altered to better define ever use: “current and/or prior use of tobacco; current and/or prior use of alcohol.”

-Dietary assessment- The authors chose poor methods of dietary assessment. It is a literature consensus that FFQ associated with 24 dietary recall is not appropriate to analyze food consumption. The more recent recommendation suggests using a 3-day food diary to explore the food consumption in the population.

Our review of the literature did not demonstrate a consensus supporting the statement “FFQ associated with 24 dietary recall is not appropriate to analyze food consumption.” However, we were able to find an article from the United States agency National Institutes of Health (DOI=10.1016/j.jand.2015.08.016) which states: “Even though there is bias in 24-hour recalls, because food records potentially have reactivity bias, the 24-hour recall is considered the least biased of the self-report instruments and the best single dietary assessment instrument for many purposes.” To that end, at least two validation papers have been published on the AHS-2 thus far, supporting the use of 24-hour recalls with a food frequency questionnaire: DOI=10.1017/S1368980009992072 and DOI=10.3945/jn.115.225508. Additionally, the AHS-2 has been conducted since 2002 and the authors of the present manuscript are unable to modify the protocols of the prospective cohort described in this manuscript.

- Dietary pattern- I could not understand if all participants are vegetarian or not. I suggest clarifying participants' dietary patterns.

We clarified by adding a new sentence in the Methods section to describe study participants’ dietary patterns: “Within this final analytical group, 37% were either vegan or lacto-ovo vegetarian, and 16% were either pesco-vegetarian or semi-vegetarian.”

- It is unclear which nutrients were supplemented or not. It made me a bit confused.

To improve clarity, we added a new sentence in the Methods section to list which nutrients were included from supplements: “Supplemented nutrients included saturated fatty acids, mono-unsaturated fatty acids, poly-unsaturated fatty acids, fiber, sodium, vitamin C, alpha-tocopherol equivalents, and flavonoids.”
